# Evaluation of squamous cell carcinoma antigen 1 expression in oral squamous cell carcinoma (tumor cells and peritumoral T-lymphocytes) and verrucous carcinoma and comparison with normal oral mucosa

**DOI:** 10.1590/1678-7757-2021-0374

**Published:** 2021-12-01

**Authors:** Samira Derakhshan, Arvin Poosti, Amirnader Emami Razavi, Mohammad Amin Moosavi, Nazanin Mahdavi, Fereshteh Baghaei Naieni, Kambiz Kamyab Hesari, Amirsina Rahpeima

**Affiliations:** 1 Tehran University of Medical Sciences School of Dentistry Oral and Maxillofacial Pathology Department Tehran Iran Tehran University of Medical Sciences, School of Dentistry, Oral and Maxillofacial Pathology Department, Tehran, Iran.; 2 Tehran University of Medical Sciences School of Dentistry Tehran Iran Tehran University of Medical Sciences, School of Dentistry, Tehran, Iran.; 3 Tehran University of Medical Sciences Cancer Institute of Iran Iran National Tumor Bank Tehran Iran Tehran University of Medical Sciences, Cancer Institute of Iran, Iran National Tumor Bank, Tehran, Iran.; 4 National Institute of Genetic Engineering and Biotechnology Institute of Medical Biotechnology Molecular Medicine Department Tehran Iran National Institute of Genetic Engineering and Biotechnology (NIGEB), Institute of Medical Biotechnology, Molecular Medicine Department, Tehran, Iran.; 5 Tehran University of Medical Sciences Razi Hospital Dermatopathology Department Tehran Iran Razi Hospital, Tehran University of Medical Sciences, Dermatopathology Department, Tehran, Iran.

**Keywords:** Oral cancer, Antigen, Lymphocyte, Squamous cell, Verrucous

## Abstract

**Background:**

Squamous cell carcinoma antigen (SCCA) is used as a prognostic marker for recurrence of squamous cell carcinoma in various sites, including head and neck. Studies suggest that its high serum levels are correlated to some clinical features, such as nodal metastasis. However, it is still unknown if high SCCA in patients with SCCA tissue expression in tumor cells are related to peripheral T-lymphocytes. Therefore, we did this study to evaluate SCCA expression in squamous cell carcinoma and verrucous carcinoma and to compare it with normal oral mucosa, also investigating the correlation between serum-based and tissue-based antigen levels.

**Methodology:**

In this study, the immunohistochemistry (IHC) technique was used to determine the SCCA1 expression pattern in 81 specimens divided into 3 groups, including oral squamous cell carcinoma, verrucous carcinoma, and normal oral mucosa. Serum-based and tissue-based antigen levels of 20 oral squamous cell carcinoma cases were compared by the western blot assay. SCCA expression was also evaluated and compared in both tumor cells and peripheral T-lymphocytes by the immunofluorescence assay.

**Results:**

Our results showed that the SCCA levels in SCC specimens were significantly lower than in verrucous carcinoma and normal and hyperplastic oral mucosa specimens. We found no correlation between the IHC expression of SCCA and serum levels. SCCA was well expressed in both tumor cells and peripheral T-lymphocytes.

**Conclusion:**

Decreasing SCCA in SCC specimens suggested that SCC tumor cells may affect more than the serum levels of SCCA in some patients. In addition, expression of SCCA in peripheral T-lymphocytes showed that both tumor cells and T-lymphocytes may cause serum SCCA.

## Introduction

Oral squamous cell carcinoma (OSCC) is the most common malignancy of the head and neck (excluding non-melanoma skin cancer).^1^ Despite progress in research and therapy, its 5-year survival rate remained the same in the last 45 years^2^ because of delayed clinical detection, poor prognosis, expensive therapeutic alternatives, and lack of specific biomarkers for the disease.^3^ Biomarkers are biological molecules found in blood, other body fluids, or tissues that indicate normal biological processes, pathogenic processes, or a pharmacological response to a therapeutic intervention.^4^

One of these markers, called squamous cell carcinoma antigen (SCCA), was found in human cervical squamous cell carcinoma tissue for the first time by Kato and Torigoe^5^ (1977). Patients with squamous cell carcinoma of the cervix or other origins, such as nasopharynx and bronchus, head and neck, healthy volunteers and patients with different tumors showed high serum levels of SCCA.^6,7^

SCCA belongs to the serine proteinase inhibitor (serpin) superfamily.^8^ *SCCA1* and *SCCA2* are two nearly identical genes, responsible for SCCA expression.^9^

SCCA mRNA can be detected in patients with OSCC and patients with non-SCC cancers, but it is relatively low in non-SCCs.^10^ Regarding tumor aggression, high mRNA levels of SCCA are characteristic to less aggressive head and neck SCC tumors.^11^

Studies showed that immunohistochemical concentration of SCCA in SCC tissues and in normal squamous epithelial was much higher than in non-SCC cancer tissues.^10,12^ Among patients with SCC, cancer cells in stage I and II show significantly higher levels of SCCA1 than in stage III and IV.^13^ SCCA1 and SCCA2 also co-express in suprabasal layers of the stratified squamous epithelium of the tongue, tonsil, esophagus, uterine cervix and vagina, Hassall’s corpuscles of the thymus, and some areas of the skin.^14^ In patients with benign diseases of the skin, SCCA expression and serum levels can be high.^15,16^ Therefore, we conducted this study to clarify expression differences between oral squamous cell carcinoma, verrucous carcinoma, and normal oral mucosa, and to investigate the association between serum-based and tissue-based expression of SCCA. The relationship between SCCA expression in tumor cells and in peripheral T-lymphocytes was also evaluated to determine the origin of the high serum levels in this antigen.

## Methodology

### Patients and tissue specimens

For this purpose, 81 formalin-fixed paraffin-embedded blocks from incisional or excisional biopsies including 27 oral squamous cell carcinomas (OSCC), 27 verrucous carcinomas (VC), and 27 normal oral mucosa specimens with only benign hyperplasia (BH) were examined. These blocks were collected from the patients referred and treated at the Razi Hospital, affiliated to the Tehran University of Medical Sciences (TUMS), Tehran City (Tehran Province, Iran). Plasma samples of 20 patients with OSCC were obtained from the Tumor Bank, Cancer Institute to compare tissue-based and serum-based antigen levels. Normal oral mucosa and benign hyperplasia specimens were taken from the epithelium of reactive lesions, such as pyogenic granuloma or irritation fibroma with no evidence of dysplasia.

This study was done with two undergraduate theses under supervision and support of the Tehran University of Medical Sciences, School of Dentistry with grant numbers #97-03-69-39741 and #98-02-69-42938. The study was approved by the Ethics Committee of the Tehran University of Medical Sciences, Dental School with ethics codes of (IR.TUMS.DENTISTRY.REC.1397.145) and (IR.TUMS.DENTISTRY.REC.1398.179).

### Immunohistochemistry and evaluation

Immunohistochemistry (IHC) was applied on paraffin blocks of 27 oral squamous cell carcinomas, 27 verrucous carcinomas, and 27 normal mucosa tissues after being cut off in sections 4 µ thick. Single antibody IHC method was done after deparaffinization and dehydration of tissues with graded ethanol, as reported previously.^17^ Tris-EDTA buffer (pH=8) was used as an antigen retrieval technique at 100°C for 35 min. To reduce temperature, slides were put at room temperature for 15 min. Hydrogen peroxide was used as a blocking agent to inactivate endogenous peroxidase. Primary antibody was incubated for 1.5 h at room temperature using Rabbit polyclonal SCC antibody or Anti-SerpinB3 antibody (ABCAM, Cambridge, Code #ab186842, 1:200). The sections were washed and counterstained lightly with hematoxylin. Squamous carcinoma specimen of the cervix was applied using PBS (phosphate-buffered saline) as positive controls, while the primary antibody was omitted using PBS as negative controls. All the slides were evaluated by two blinded oral pathologists using an Olympus BX51 microscope. Diffuse cytoplasmic brown immunoreaction was described as positive staining. Observers considered faint diffuse or single nuclear and stromal inflammatory cell staining as negative staining. IHC-scoring scale, including staining percentage of tumor cells (0-100%) with SCCA expression in random 10 high-power fields, measured as follows:

Score 0: no expression; Score 1: less than 10% of tumor cells; Score 2: 10-50% of tumor cells; Score 3: more than 50% of tumor cells.^18^

### Western blotting

A total of 20 plasmas with 20 formalin-fixed paraffin-embedded blocks were obtained from patients with OSCC (Table 1). Human plasma samples were first emptied from albumin and enriched with low-abundant proteins by trichloroacetic acid (TCA)/acetone method as described elsewhere.^19^ Then, protein concentrations were measured by the Bradford assay, and an equal number of proteins (40-60 μg) was subjected to sodium dodecyl sulfate polyacrylamide gel electrophoresis (SDS-PAGE), being transferred onto nitrocellulose membranes (Whatman, UK). Later, the membranes were blocked by non-fat dry milk powder (5%, v/v) in TBST (Tris-buffered saline and 0.05% Tween-20) for 1 h at room temperature (RT) and then incubated with anti-SerpinB3 antibody (Code #ab186842 purchased from ABCAM, Cambridge, 1:200) overnight at 4°C. After three washes with 0.2% TBST, the membranes were incubated with a horseradish peroxidase-conjugated secondary antibody (1:10000, purchased from Sigma-Aldrich, Germany) for 2h at RT. Finally, protein bands were detected by an enhanced chemiluminescence kit (Amersham Life Sciences, UK) on X-ray.

**Table 1:** Characteristics of the plasma samples

Number	Sample Number	Storage Address	Type	Diagnosis	Prep
1	A00344107	FF1-R06-T05-030	PLASMA	OSCC	FF
2	A00361107	FF2-R01-B01-089	PLASMA	OSCC	FF
3	A00972107	FF3-R02-T06-088	PLASMA	OSCC	FF
4	A01001110	FF3-R02-T09-034	PLASMA	OSCC	FF
5	A01004107	FF3-R02-T09-058	PLASMA	OSCC	FF
6	A01154107	FF3-R04-T05-058	PLASMA	OSCC	FF
7	A01162107	FF3-R04-T06-010	PLASMA	OSCC	FF
8	A01240107	FF1-R01-T02-044	PLASMA	OSCC	FF
9	A01251107	FF2-R03-B05-090	PLASMA	OSCC	FF
10	A01254107	FF2-R04-B03-043	PLASMA	OSCC	FF
11	A01286111	FF1-R02-T04-068	PLASMA	OSCC	FF
12	A01396111	FF3-R05-T03-080	PLASMA	OSCC	FF
13	A01472111	FF1-R01-T02-091	PLASMA	OSCC	FF
14	A01591111	FF2-R01-B05-020	PLASMA	OSCC	FF
15	A01593107	FF2-R01-B07-014	PLASMA	OSCC	FF
16	A01777111	FF4-R02-T05-094	PLASMA	OSCC	FF
17	A01816121	FF4-R03-T03-036	PLASMA	OSCC	FF
18	A01863111	FF1-R01-T03-015	PLASMA	OSCC	FF
19	A01865111	FF4-R04-T01-097	PLASMA	OSCC	FF
20	A01951108	FF4-R05-T01-089	PLASMA	OSCC	FF

### Immunofluorescence

A total of 10 specimens of moderately different oral squamous cell carcinoma with moderate inflammatory cell infiltration was used to compare SCCA expression in tumor cells and in peripheral T-lymphocytes. Inflammation of cells in connective tissue around the tumor was evaluated as previously described.^20^ Briefly, 4 μ sections were deparaffinized twice with xylol for 10 min and hydrated with water for 3 min. Antigen retrieval was done using 2H2O/CaCL2 at 37°C, followed by RT for 20 min and 10 min respectively, and washed twice by PBS-T (0.05%). Membranous permeability was increased using Triton (0.5%), then blocked with a secondary antibody (1:10 dilution) for 1 h at 37°C, and washed twice by PBS-T. Incubation with anti-serpinB3/SCCA and anti-CD3 primary antibodies (1:200, rabbit polyclonal, ABCAM, #ab154971 and 1:200, mouse monoclonal, ABCAM, #ab699) was conducted for 2 h at 37°C. After three washes with PBS, specimens were incubated with fluorescent secondary antibodies (1:400, goat anti-rabbit, IgG H&L, Alexa flour-488, #ab150077 and 1:400, goat anti-mouse IgG H&L, Alexa flour-647, #ab150115) for 2 h at 37°C. After two washes with PBS-T for 7 min, DAPI123 was used for nuclear staining for 1 min and, finally, mounting fluid was applied at 4°C.

Two investigators evaluated staining alone. Specimens were considered positive for SCCA when 5% of the cells showed diffuse cytoplasmic green staining for SCCA and red staining for CD3.^21^

### Statistical analysis

IBM SPSS statistical software version 23.0 (IBM Corporation, Armonk, NY, USA) was used to analyze the data. The Mann-Whitney U and Fisher’s tests were applied to correlate the tissue-based and serum-based antibody expression. Kruskal-Wallis test was used to investigate the relationship between groups of lesions. P-value ≤0.05 was considered statistically significant.

## Results

### Immunohistochemistry

**Figure 1:**
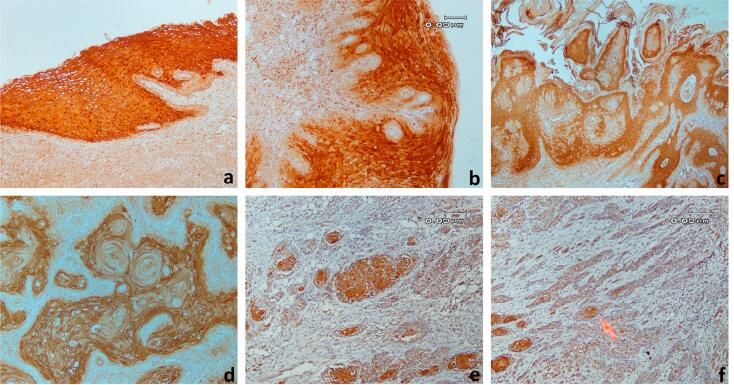
Immunohistochemistry results a) Strong positive normal keratinocytes b) Strong positive epithelial cells in benign epithelial hyperplasia c) Strong positive epithelial cells in verrucous carcinoma d) Strong positive reaction in more than 50% of the tumor cell (score 3) e) Strong positive reaction in 10-50% of tumor cells (score 2) f) Strong positive reaction in less than 10% of tumor cells (score 1)

**Figure 2:**
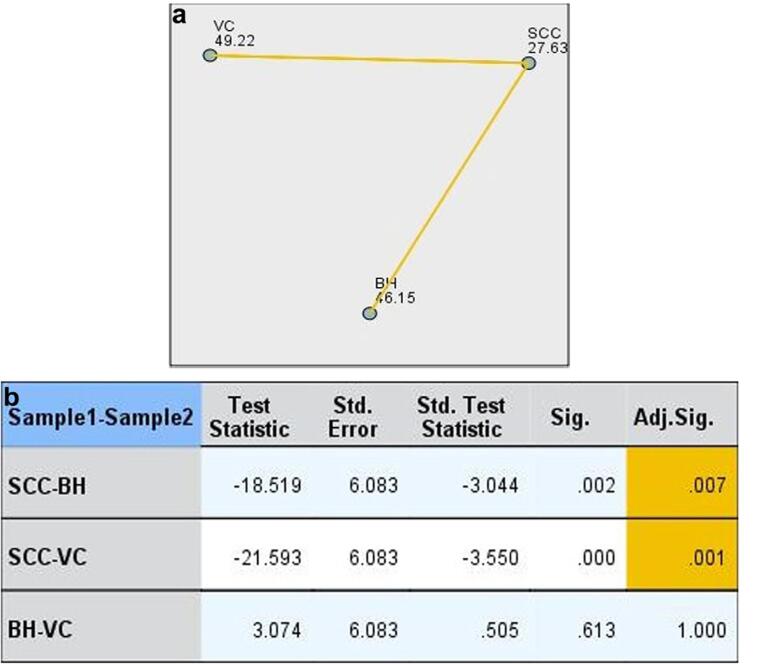
Pairwise comparison of diagnosis between SCC, VC and BH a) each node shows the average rank of diagnosis for samples b) each row tests the null hypothesis that sample 1 and sample 2 have the same distributions. Asymptomatic significance (2-sided tests) is displayed. The significance level is 0.05. Significance values have been adjusted by the Bonferroni correction for multiple tests

We observed SCCA immunostaining in the cytoplasm of typical epithelial, verrucous carcinoma, cancer cells and T-lymphocytes peripheral to cancer tissue (Figure 1). Among 27 primary SCC specimens, 10 did not express SCCA (37%). Ten patients expressed SCCA in less than 10% of total cells (37%). Only 7 out of 27 cases expressed SCCA in more than 10% of the total cancer cells (22.2%: 10-50% of cells or score 2 and 3.7%: more than 50% of cells or score 3) (Table 2). Sixteen verrucous carcinoma specimens had scores 2 and 3 (both 29.6%), and 10 cases had score 1 (37%) for SCCA expression. Fifteen benign hyperplasia specimens had scores 2 and 3 (40.7% and 14.8%, respectively), and 12 cases had score 1 (44.4%) for SCCA expression. Only one case of verrucous carcinoma had score 0. No case of benign hyperplasia had score 0. SCC specimens expressed significantly less SCCA than verrucous carcinoma and benign hyperplasia specimens (p=0.001 and p=0.007, respectively) (Figure 2).

**Table 2:** Comparison of 81 samples in 4 ranges of SSCA IHC level and divided into 3 groups: 27 squamous cell carcinomas (SCC) 27 verrucous carcinomas (VC) and 27 benign hyperplasia (BH)

Diagnosis	0	0-10	0-50	50-100	Total
**SCC**					
Count	10.00	10	6	1	27
% within Diagnosis	37.00%	37.00%	22.2%	3.7%	100,00%
% within SCCA IHC level	90.9%	31.3%	24.00%	7.7%	33.3%
Adjusted Residual	4.4	-0.3	-1.2	-2.1	
**VC**					
Count	1.00	10	8	8	27
% within Diagnosis	3.7%	37.00%	29.6%	29.6%	100,00%
% within SCCA IHC level	9.1%	31.3%	32.00%	61.5%	33.3%
Adjusted Residual	-1.8	-0.3	-0.2	2.4	
**BH**					
Count	0.00	12	11	4	27
% within Diagnosis	0.00%	44.4%	40.7%	14.8%	100,00%
% within SCCA IHC level	0.00%	37.5%	44.00%	30.8%	33.3%
Adjusted Residual	-2.5	0.6	1.4	-0.2	
**Total**					
Count	11.00	32	25	13	81
% within Diagnosis	13.6%	39.5%	30.9%	16.00%	100,00%
% within SCCA IHC level	100.00%	100.00%	100.00%	100,00%	100,00%

### Western blot analysis

**Figure 3:**
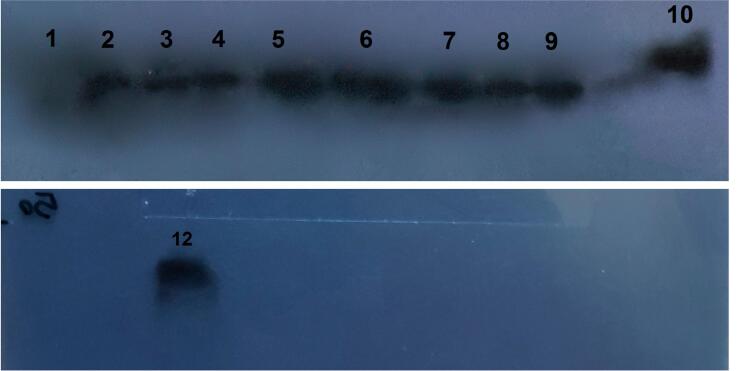
Western blot shows positive results for samples 2 to 10 and sample 12 (Numbers are mentioned in Table 3)

Western blot analysis revealed 10 cases of positive SCCA in SCC plasmas, 9 cases of negative SCCA, and one technical error (Table 3) (Figure 3). There was no correlation between the IHC analysis of tissue-based protein and the western blot analysis of serum-based protein (p=0.71 and p=0.83 in two separate analyses) (Table 4).

**Table 3:** Comparison of western blot analysis of plasma and IHC analysis of tissue-based samples

Number	Sample number	IHC	Western blot	Type
1	A01162107	50-100%	error	SCC
2	A00361107	50-100%	positive	SCC
3	A00344107	50-100%	positive	SCC
4	A00972107	50-100%	positive	SCC
5	A01816121	10-50%	positive	SCC
6	A01154107	10-50%	positive	SCC
7	A01593107	10-50%	positive	SCC
8	A01286111	0-10%	positive	SCC
9	A01951108	0-10%	positive	SCC
10	A01254107	0-10%	positive	SCC
11	A01001110	50-100%	negative	SCC
12	A01396111	0-10%	positive	SCC
13	A01472111	10-50%	negative	SCC
14	A01591111	0-10%	negative	SCC
15	A01240107	0-10%	negative	SCC
16	A01777111	50-100%	negative	SCC
17	A01004107	50-100%	negative	SCC
18	A01863111	0-10%	negative	SCC
19	A01865111	50-100%	negative	SCC
20	A01251107	0-10%	negative	SCC

**Table 4:** Correlation between IHC analysis and western blot analysis

IHC	W.B		
**0-10%**	**Negative**	**Positive**	**Total**
Count	4	4	8
% within IHC	50.00%	50.00%	100.00%
% within W.B	44.4%	40.00%	42.1%
**10-50%**			
Count	1	3	4
% within IHC	25.00%	75.00%	100.00%
% within W.B	11.1%	30.00%	21.1%
**50-100%**			
Count	4	3	7
% within IHC	57.1%	42.9%	100.00%
% within W.B	44.4%	30.00%	36.8%
**Total**			
Count	9	10	19
% within IHC	47.4%	52.5%	100.00%
% within W.B	100.00%	100.00%	100.00%

### Immunofluorescence

**Figure 4:**
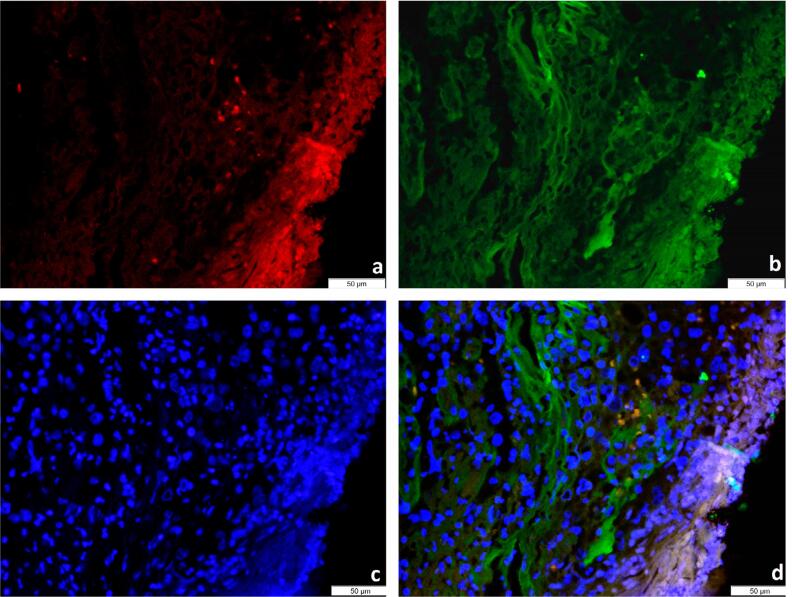
Immunofluorescence results a) Positive reaction for CD3 b) Positive reaction for SCC-antigen c) DAPI staining showed cell nuclei d) Co-expression of CD3 and SCC-antigen in more than 5% of the cells

Immunofluorescence results showed SCCA immunostaining in T-lymphocytes (CD3 positive cells) in all 10 specimens (Figure 4). Two investigators observed SCCA immunoreaction in tumor cells in 8 out of 10 specimens. SCCA expression showed no significant differences in tumor cells and peripheral T-lymphocytes. (p=0.44).

## Discussion

Our results revealed that SCCA1 expression was significantly higher in hyperplastic epithelium and verrucous carcinoma than in OSCC (p=0.001 and p=0.007, respectively). We also found that SCCA1 serum levels were not related to tissue expression (p=0.71 and p=0.83 in two separate analyses).

Based on the above, we designed an experiment to determine origin cells with SCCA1 protein and found that peripheral T-lymphocytes produce more SCCA1 than tumor cells, but the SCCA1 expression is the same in both.

A biomarker is a substance that can help oncologists in the diagnosis, prognosis, and follow-ups of diseases. Biomarkers are mostly secreted from tumors, but some can be a body response to cancer. However, they are not specific and sensitive enough to detect cancers with precision.^4^ A lack of specific biomarkers for SCC is the main obstacle to improve survival rates.^2,3^

Several studies reported more SCCA1 than SCC in normal epithelium and benign epithelial lesions. Murakami, et al.^22^ (2000) reported lower SCCA1 at mRNA level in cervical SCC than in the normal cervical tissue.Barnes, Coulter and Worrall^23^ (2000) found a lower SCCA1 production in cases with cervical squamous cell cancer than in those with a healthy cervix. Yasumatsu, et al.^21^ (2001) found that normal keratinocytes had more SCCA1 than head and neck SCC cells, and that the SCCA1 level was significantly higher than the sinonasal SCC level in both tissue and serum expression in inverted papilloma.^24^To the best of our knowledge, this study is the first to compare SCCA1 in verrucous carcinoma and benign hyperplasia of oral epithelium with SCCA1 in oral SCC.

Travassos, et al.^25^ (2018), in their review study, demonstrated that high serum levels of SCCA as a biomarker were correlated with an advanced TNM (tumor, nodes, metastases) stage of head and neck SCC, being higher in stage III and IV than in stage I and II. Previous studies reported increased SCCA2/SCCA1 mRNA ratio in SCC cancer. Correlation between the SCCA2/SCCA1 mRNA ratio and tumor stage and cancer recurrence was also found. Stenman, et al.^26^ (2001) found that in head and neck SCC the SCCA2/SCCA1 ratio increased following tumor progression. From these findings, one can conclude that SCCA1 serum levels increase; however, from our results, tissue expression is not associated with serum levels.

While the cause of SCCA1 in patients with SCC remains controversial, previous studies suggested that SCC tumor cells increase SCCA in a patient’s serum. Therefore, tumor size could influence serum levels of SCCA1. However, Travassos, et al.^25^ (2018) proved this wrong. Uemura, et al.^27^ (2000) suggested that active secretion to medium culture does not cause SCCA1 in serum, but a passive release increases serum levels of SCCA1 in advanced squamous cell carcinoma. Cataltepe, et al.^14^ (2000) found SCCA1 mRNA in normal lymph nodes by the reverse transcription-polymerase chain reaction (RT-PCR), but it is still impossible to do so by IHC assay. Based on previous studies, lymph node metastasis influences serum levels of SCCA1 in tumor size, even in early stages of cervical SCC.^28^ Our results confirmed findings of Yasumatsu, et al.^21^ (2001), suggesting that peripheral T-lymphocytes increase SCCA1 serum; however, we found that, besides T-lymphocytes, tumor cells can also express SCCA1. On the other hand, Schaik, et al.^29^ (2019) reported that SCC-Ag concentration in fine-needle aspiration specimens was higher in lymph nodes of patients with head and neck SCC than in other lymph nodes (p<0.01). They introduced this finding as a new method to detect cervical lymph nodes metastases in patients with squamous cell carcinoma of the head and neck. This could match with our results, since if T-lymphocytes can produce SCCA, lymph nodes with micrometastases can detect SCCA rather than diagnose metastatic squamous cells in biopsy specimens or clinical examinations like computer tomography (CT) scan.

Further studies suggest correlating TNM stage, human papillomavirus (HPV) status, and tumor site with serum-based and tissue-based levels of SCCA1 in patients with SCC, besides using serum levels of SCCA2 and total SCCA and tissue expression to investigate how SCCA2 influences progression and aggression of head and neck SCC.

## Conclusion

In conclusion, our findings suggested that serum levels of SCCA1 are not associated with tissue-based expression. Decreasing SCCA in SCC specimens showed that SCC tumor cells may affect more than serum levels of SCCA in some patients. Finally, immunofluorescence results showed that both tumor cells and peripheral T-lymphocytes can express and produce SCCA1, causing serum SCCA.
